# *Chlamydia muridarum* Can Invade the Central Nervous System *via* the Olfactory and Trigeminal Nerves and Infect Peripheral Nerve Glial Cells

**DOI:** 10.3389/fcimb.2020.607779

**Published:** 2021-01-08

**Authors:** Lynn Nazareth, Heidi Walkden, Anu Chacko, Ali Delbaz, Todd Shelper, Charles W. Armitage, Ronak Reshamwala, Logan K. Trim, James A. St John, Kenneth W. Beagley, Jenny A. K. Ekberg

**Affiliations:** ^1^ Menzies Health Institute Queensland, Griffith University, Southport, QLD, Australia; ^2^ Clem Jones Centre for Neurobiology and Stem Cell Research, Griffith University, Nathan, QLD, Australia; ^3^ Centre for Immunology and Infection Control, School of Biomedical Sciences, Queensland University of Technology, Brisbane, QLD, Australia; ^4^ Griffith Institute for Drug Discovery, Griffith University, Nathan, QLD, Australia

**Keywords:** Chlamydia, olfactory ensheathing cell, Schwann cell, olfactory bulb, cytokine, amyloid, bacteria

## Abstract

*Chlamydia pneumoniae* can infect the brain and has been linked to late-onset dementia. *Chlamydia muridarum*, which infects mice, is often used to model human chlamydial infections. While it has been suggested to be also important for modelling brain infection, nervous system infection by *C. muridarum* has not been reported in the literature. *C. pneumoniae* has been shown to infect the olfactory bulb in mice after intranasal inoculation, and has therefore been suggested to invade the brain *via* the olfactory nerve; however, nerve infection has not been shown to date. Another path by which certain bacteria can reach the brain is *via* the trigeminal nerve, but it remains unknown whether *Chlamydia* species can infect this nerve. Other bacteria that can invade the brain *via* the olfactory and/or trigeminal nerve can do so rapidly, however, whether *Chlamydia* spp. can reach the brain earlier than one-week post inoculation remains unknown. In the current study, we showed that *C. muridarum* can within 48 h invade the brain *via* the olfactory nerve, in addition to infecting the trigeminal nerve. We also cultured the glial cells of the olfactory and trigeminal nerves and showed that *C. muridarum* readily infected the cells, constituting a possible cellular mechanism explaining how the bacteria can invade the nerves without being eliminated by glial immune functions. Further, we demonstrated that olfactory and trigeminal glia differed in their responses to *C. muridarum*, with olfactory glia showing less infection and stronger immune response than trigeminal glia.

## Introduction

The bacterium *Chlamydia pneumoniae*, which causes pneumonia, can also infect the central nervous system (CNS). Several studies have linked *C. pneumoniae* infection to Alzheimer’s disease (AD), in particular late-onset AD, now typically termed late-onset dementia (reviewed in Balin et al., 2018). *C. pneumoniae* DNA has been detected in significantly more post-mortem brains from late-onset dementia patients than from age-matched controls (80–90 *versus* 5–10%) (Balin et al., 1998; Gerard et al., 2006). Viable *C. pneumoniae* organisms have also been isolated from post-mortem late-onset dementia patient brains (Balin et al., 2018) and *C. pneumoniae* antigens have been detected next to or within senile plaques in these brains (Balin et al., 1998; Gerard et al., 2006; Hammond et al., 2010). Some studies, however, have not detected any difference in the amount of *C. pneumoniae* DNA between post-mortem brains of patients with late-onset dementia and control brains (Ring and Lyons et al., 2000; Taylor et al., 2002).

Several studies in mice have shown that intranasal inoculation with *C. pneumoniae* can lead to accumulation of amyloid β (Aβ), a key hallmark of late-onset dementia/AD, in the cerebral cortex (Little et al., 2004; Boelen et al., 2007; Little et al., 2014). Significantly, these studies were conducted in wild-type mice, suggesting that *C. pneumoniae* can cause pathology without an underlying genetic predisposition. However, *C. pneumoniae* is a human pathogen which can be difficult to culture in the laboratory. Modelling of chlamydial genital and lung infections in mice is therefore often performed using *Chlamydia muridarum*, which infects rodents and also exhibits fast growth kinetics and high yield (Skelding et al., 2006; Patel et al., 2015). Furthermore, to establish lung infection in mice, *C. muridarum* requires a significantly lower inoculation dose than *C. pneumoniae* (Horvat et al., 2007; Woods et al., 2020) and for genital tract infection it requires far less inoculation dose that *C. trachomatis* (Carey et al., 2009). *C. muridarum* has also been suggested to be useful for investigating CNS infection by *Chlamydia* spp. in mice and rats (Balin et al., 2018; Woods et al., 2020), but has not been described in the literature.

**Figure 1:**
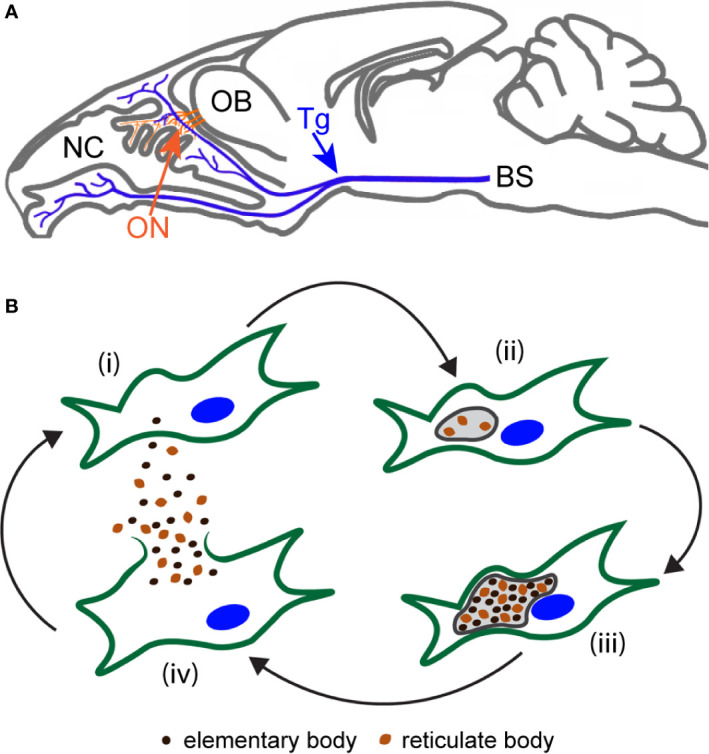
Anatomy of the olfactory/trigeminal nerves and CNS in mice, and the *C. muridarum* lifecycle. **(A)** The schematic shows a sagittal view of the nasal cavity (NC) with the olfactory nerve (ON; orange), which terminates in the olfactory bulb (OB), and the trigeminal nerve (Tg, blue), which terminates in the brainstem (BS). **(B)** A schematic showing the lifecycle of *C. muridarum* within a cell (green with nucleus in blue). (i) Cells are infected by extracellular elementary bodies (EB; dark brown). (ii) Within the inclusion (grey), the EBs develop into reticulate bodies (RB; light brown) and (iii) replication leads to large inclusions. (iv) Towards the end of the lifecycle, RBs condense into infectious EBs. Cell lysis then releases infective EBs to infect other cells.

What also remains unknown is how *Chlamydia* species (spp.) can reach the brain. Since *Chlamydia* spp. can infect lung macrophages, one possible mechanism is *via* blood-borne macrophages followed by migration across the blood-brain barrier (Gieffers et al., 2004). Invasion *via* the olfactory nerve has also been strongly suggested, as *C. pneumoniae* bacteria were detected in the olfactory bulb of mice following intranasal inoculation (Boelen et al., 2007; Little et al., 2014). The olfactory nerve connects the nasal epithelium with the brain, as do the intranasal branches of the trigeminal nerve (**Figure 1A**). Interestingly, the brain regions where these two nerves merge with the CNS (the olfactory bulb and the brainstem, respectively) are the first to show signs of pathology in AD (Mann et al., 1988; Christen-Zaech et al., 2003; Murphy, 2019).

Another key aspect of infection of the CNS by *Chlamydia* spp. that remains to be determined is how fast the bacteria can reach the CNS following intranasal inoculation. The earliest time-point at which *C. pneumoniae* has been detected within the olfactory bulb and cerebral cortex following intranasal inoculation in mice is 7 days (Boelen et al., 2007). We have shown that another bacterium, *Burkholderia pseudomallei*, can rapidly (within 24–48 h) invade the brain *via* the olfactory and trigeminal nerves, without blood infection (Dando et al., 2014; St John et al., 2014; Dando et al., 2016; St John et al., 2016). *Streptococcus pneumoniae* (Rake, 1937), *Neisseria meningitidis* (Sjolinder and Jonsson et al., 2010), and *Staphylococcus aureus* (Herbert et al., 2012) can also rapidly infect the brain (olfactory bulb) *via* the olfactory nerve. Thus, it is possible that *Chlamydia* spp. may reach the CNS much faster than one week post intranasal exposure.

*Chlamydia* spp. are Gram negative, obligate intracellular bacteria, and must thus replicate inside host cells. The bacteria have a unique biphasic life-cycle (**Figure 1B**). The first part of the cycle is the extracellular infectious elementary body (EB) stage. Once inside host cells, EBs become encapsulated within an inclusion body, in which the EB converts into the second phase, the metabolically active, replicating, reticulate body (RB) (Maxion et al., 2004). Replication continues until the life-cycle is completed, after which the RBs convert into infectious EBs, which are released from the host cells *via* lysis or extrusion (Zuck et al., 2016). Whilst it is not fully known which cells are the hosts of *Chlamydia* spp. in the nervous system, *C. pneumoniae* bacteria have been found inside glial cells (astrocytes and microglia) in post-mortem human brain tissue (Balin et al., 1998; Gerard et al., 2006; Hammond et al., 2010), showing that the bacteria can infect at least some types of glial cells.

One key mechanism thought to enable certain bacteria to reach the CNS after intranasal inoculation is the ability to infect the glial cells of the olfactory and trigeminal nerves (Dando et al., 2014). The glia of the olfactory nerve, olfactory ensheathing cells (OECs), are the main phagocytes of the olfactory nerve (Su et al., 2013; Nazareth et al., 2015). OECs can internalize bacteria and mount a powerful innate immune response to the pathogens (Vincent et al., 2007; Harris et al., 2009; Herbert et al., 2012; Panni et al., 2013). The glia of the trigeminal nerve are trigeminal Schwann cells (TgSCs), which can also internalize bacteria (Panni et al., 2013). Schwann cells, however, have been shown to exhibit a significantly lower immune response to pathogens than OECs (Vincent et al., 2007). Some of the bacteria that can invade the olfactory/trigeminal nerves have been shown to infect rather than be cleared by OECs/TgSCs, including *S. pneumoniae* (Macedo-Ramos et al., 2016), *B. pseudomallei* (Walkden et al., 2020), and *N. meningitidis* (Delbaz et al., 2020). Thus, one possibility by which *Chlamydia* spp. may be able to invade the CNS is by infecting these glia.

In the current study, we investigated whether *C. muridarum* could invade the CNS after intranasal inoculation in mice. We focussed on short-term (48 h) infection and the nose-to-brain nerve route. We also investigated whether *C. muridarum* could infect and remain viable within cultured OECs and TgSCs, and how the glia responded to *C. muridarum* by secretion of cytokines and chemokines.

## Methods

### Ethics

All procedures were approved by Griffith University’s Animal Ethics Committee (ESK/02/15/AEC and MSC/08/18/AEC) under the guidelines of the Australian Commonwealth Office of the Gene Technology Regulator.

### *Chlamydia muridarum* Culture

*C. muridarum* Weiss strain isolates (ATCC VR-123, VA, USA) used for this study were propagated in McCoy B cells (Andrew et al., 2013).

### Mice and Intranasal Inoculation With *Chlamydia muridarum*

For intranasal inoculations, mice were lightly anesthetized with 5% isoflurane (1 L/min) to facilitate inhalation of the entire inoculum. Inoculum was prepared in 10 µl aliquots in Hank’s balanced salt solution (HBSS) delivered as a 5 µl droplet per nostril. For organ load and immunohistochemistry experiments, adult BALB/c mice were inoculated with *C. muridarum* at high dose (1x10^6^ inclusion forming units (IFU); N = 14) or low dose (1 x 10^3^ IFU; N = 14). Control mice were inoculated with HBSS (vehicle) alone. Inoculations were performed across two weeks (7 high dose-inoculated, 7 low dose-inoculated and either 1 or 2 control mice per week (N = 3 total). For some experiments (immunohistochemistry of tissues only) adult S100β-DsRed mice were intranasally inoculated with either HBSS as vehicle control (N = 3) or *C. muridarum* (1 x 10^6^ IFU; N = 5). Mice were sacrificed 48 h post intranasal inoculation by rising CO_2_ asphyxiation.

### Organ Load Assays

The olfactory mucosa, olfactory bulb, brain, trigeminal nerve, and lungs were collected and placed into pre-weighed tubes containing 100 µl sucrose phosphate buffer (SPG; 250 mM sucrose, pH 7.2) and two sterile homogenization beads. Tubes were weighed again and then placed into a Qiagen TissueLyser II homogenizer. Samples were processed for 2x1 min at 26 Hz. Homogenates were then probe-sonicated for 3x20 s at 20 Hz (Sonics Vibra-Cell VCX 130). Homogenates were serially diluted onto McCoy B cell monolayers in 96-well plates, 4 h after inoculation, cell medium containing homogenized tissue was removed and then replaced with fresh cell culture medium containing cycloheximide (1 µg/ml in H_2_O, Sigma-Aldrich, C7698). After 24 h, cells were fixed using 4% PFA for 10 min at room temp. *C. muridarum* inclusions were immunolabeled as described below and counted. The number of *C. muridarum* inclusions within the whole well was counted manually at the appropriate dilution (i.e. where excessive cell death, due to homogenized tissue, was not apparent). The IFU/0.1 g tissue for each sample was calculated.

### Immunohistochemistry

Following sacrifice, heads of mice were fixed in 4% PFA overnight at 4°C, then decalcified using 20% ethylenediaminetetraacetic acid (EDTA) for 4 weeks. Heads were embedded in optimal cutting temperature (O.C.T.) medium (ProSciTech), frozen and 50 µm cryostat sections were cut in either coronal or sagittal planes. Tissue sections were blocked with 2% bovine serum albumin (BSA) in phosphate-buffered saline (PBS) with 0.3% Triton-X100 solution for 60 min at room temp. Sections were then incubated with primary antibodies raised against the major outer membrane protein (MOMP) of *C. muridarum* (sheep anti-MOMP, 1:300) (O’Meara et al., 2013), diluted in PBS-Triton-X100 overnight at 4°C, then washed and incubated with donkey anti-sheep Alexa Fluor 488 (Abcam, Ab150177) at room temp for 1 h, washed and stained with 4’,6-diamidino-2-phenylindole (DAPI, Thermofisher D1306).

### Imaging and Analysis of Tissue Sections

Tissue sections were scanned for presence of *C. muridarum* on a Nikon Eclipse Ti2-E epifluorescence microscope. High resolution images were acquired on an Olympus FV3000 laser scanning confocal microscope. Three-dimensional reconstructions were made using Imaris x64 (Version 7.4.2). For comparison between groups, the same image capture settings, laser intensity and focal depths were used. Images were color balanced uniformly across the field of view using Adobe Photoshop Creative Cloud 2018 (19.1.4) and compiled into panels using Adobe Illustrator Creative Cloud 2018 (22.1).

### Cell Culture

Primary cell cultures of glia were prepared from S100β-DsRed transgenic mice as previously described (Windus et al., 2007; Panni et al., 2013; Nazareth et al., 2019). Postnatal day 7 pups were decapitated followed by tissue dissection. For culture of OECs, the nerve fibre layer of the olfactory bulb was isolated and for TgSC culture, the trigeminal ganglia were isolated. The explants were transferred with glial cell culture medium to a 24-well plate pre-coated with 1:10 matrigel (BD Bioscience). Glia medium consisted of Dulbecco’s Modified Eagle Medium (DMEM) (Gibco), 10% FBS, G5 supplement (Gibco), gentamycin (50 μg/ml, Gibco) and L-glutamine (200 μM, Gibco). After glial cells emerged and reached confluence, they were plated for assays. Around 80% of cells obtained through this method were DsRed positive cells; we have previously shown that these DsRed positive are positive for glial markers (p75NTR, s100) (Windus et al., 2007; Panni et al., 2013; Nazareth et al., 2019). The McCoy B cell line (ATCC, CRL-1696) and the mouse monocyte/macrophage J774A.1 cell line (Ralph et al., 1975) (ATCC, TIB-67) were maintained in DMEM, 10% FBS, L-glutamine (200 μM), and gentamycin (50 μg/ml). All cells were cultured in a humidified incubator at 37°C with 5% CO_2_.

### *Chlamydia muridarum* Infection of Cultured Cells

Host cells (OECs, TgSCs, McCoy B and J774A.1 macrophage cells) were plated onto 96-well plates (growth area: 0.32 cm^2^) at 4,000 cells per well. The following day cells were infected with various multiplicities of infection (MOI) of *C. muridarum.* The infections were carried out in a static culture, accompanied by medium change after 4 h. Twenty-four hours after infection, cells were washed with PBS and fixed for 12 min using 4% PFA. *C. muridarum* inclusions were labelled using anti-MOMP. For initial experiments, and for testing of the percentage of infected cells, MOIs of 0.5:1, 1:1, 2:1, and 5:1 were used. A MOI of 2:1 was used for the following experiments, with the exception of the growth kinetics experiment which used a MOI of 0.5:1.

### Viability Assay

Host cells were infected with *C. muridarum* as described above. After 24 h, SPG with 5 mM L-glutamine was added to samples and stored at -80°C. Samples plates were thawed at 37°C and probe sonicated for 10 s, three times (Sonics Vibra-Cell VCX 130, amp 1). Cell lysates (containing bacteria) were collected and serially diluted on a monolayer of McCoy B cells and, 24 h later, washed with PBS and fixed with 4% PFA. Following immunocytochemistry, inclusions per well were counted.

### *Chlamydia muridarum* Growth Kinetics

To determine the time-course of the *C. muridarum* lifecycle, cells were infected with *C. muridarum* MOI 0.5:1 in a 96-well plate. At prescribed time points (6, 12, 24, 30, 32, 36, 40, and 48 h) SPG was added and the plates were stored at -80°C. Infectious yield at various time-points were enumerated on the McCoy B monolayer. To determine limit of detection of our imaging assay a 10-fold serial dilution series was performed for different known stocks of *C. muridarum* (2.2x10^8^ IFU/ml, 1.1x10^7^ IFU/ml, and 1.25x10^6^ IFU/ml) across a 96-well plate containing a monolayer of McCoyB cells. *C. muridarum* IBs were labelled with anti-MOMP antibody and number of bacterial inclusions per well quantified using automated image analysis software (Nikon General Analysis). IB segmentation was performed based on size and fluorescence intensity of IBs with uninfected cells used as control. The *C. muridarum* quantified from wells containing 10^5^–10^3^ IFU/ml was within range of linearity for every stock of bacteria. IFU/ml quantified from this dilution also corresponded to the original stock of bacteria.

### Immunocytochemistry

Immunocytochemistry was performed similarly to immunohistochemistry above. *C. muridarum* was detected using sheep anti-MOMP (1:500), followed by donkey anti-sheep Alexa Fluor 647 (Abcam Ab150179, 1:2,000). The nuclei of cells were stained using Hoechst 33342 (1 μg/ml, ThermoFisher Scientific). McCoy B and J774A.1 were visualized by CellMask Orange Plasma membrane stain (5 μg/ml, ThermoFischer Scientific). Cell death post infection was determined by staining with DRAQ7 (0.6 μM, Abcam).

### Cell Imaging and Analysis

Higher resolution images to study inclusions in cells were taken on an Olympus FV3000 confocal microscope. For all assays in which infection of cells was quantified, the entire wells were imaged using Nikon Eclipse Ti-2 inverted microscope and IFU per well determined. Inclusions were counted using a custom image analysis pipeline developed with the Nikon NIS-Elements General Analysis software, with the assumption that one inclusion represented one infected cell. For the higher MOIs, multiple inclusions per cell were sometimes observed and percentage of infection was determined by number of inclusions per well/initial seeding density x 100. McCoy B and J774A.1 have a doubling time of approximately 18 h, OECs and SCs a doubling time of around 100 h. However, in our doubling studies we found that both McCoy B and J774A.1 have a lag period of around 17 h post-seeding. This lag period was taken into account while performing infection. The cells were seeded late in the evening with infection early the following day. Thus the initial cell numbers when bacteria was added were similar for all cell types. To calculate infection, we used initial seeding density as the final host cell counts would be different for each cell type. The size of the inclusions during development was determined by calculating the average area of all inclusions per well using the general analysis software. Cell area was measured using Image J.

### Multiplex Assays

Cytokine and chemokine levels were determined using the Bio-Plex Pro Mouse Cytokine Standard 23-Plex, Group-1 kits from Bio-Rad. Host cells were infected at a MOI of 2:1 and supernatants collected at 3, 6, 12, 24, and 48 h post inoculation, processed as per manufacturer’s guidelines and stored at -80°C. The assay kit also included lyophilized standards which were reconstituted and diluted as per the manufacturer’s instructions and standard curves plotted. The plates were read on a BioPlex 200 Luminex bead array reader (Bio-Rad). The assay included supernatants from uninfected cells to determine the baseline level cytokine expressions in cells.

### Statistical Analyses

Statistical analyses were performed in GraphPad Prism 7; either Student’s T-tests, one-way ANOVA or two-way ANOVA with Tukey’s post-hoc test was performed. Data shown as the mean +/- the standard error of the mean (SEM).

## Results

### *Chlamydia muridarum* Can Infect the Primary Olfactory Nervous System, Trigeminal Nerve, and Brain in Mice After Intranasal Inoculation

**Figure 2:**
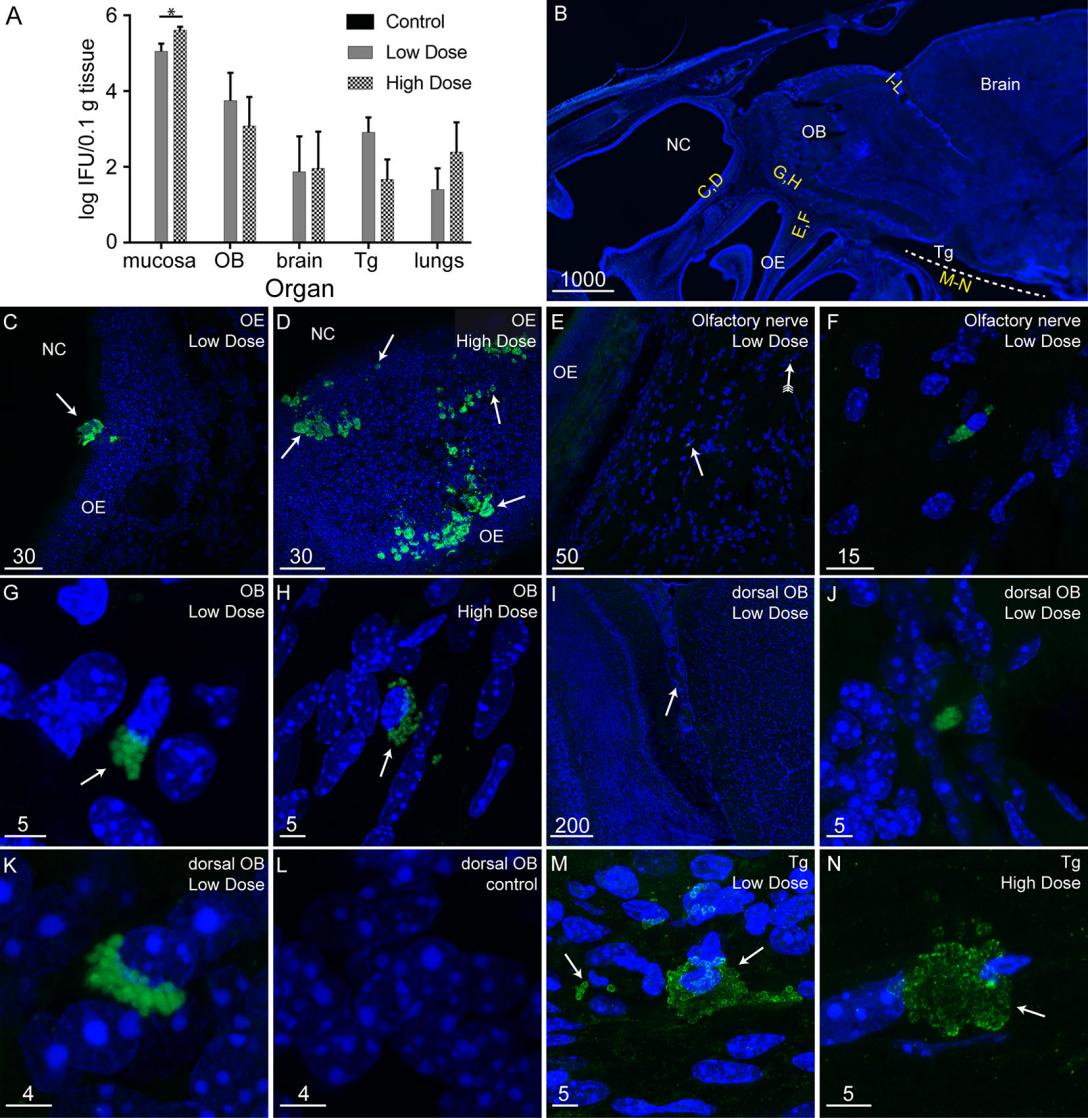
*C. muridarum* can infect the mucosa, olfactory bulb, brain and trigeminal nerve in BALB/c mice. **(A)** Graph showing the amount of infectious *C. muridarum* isolated from tissues after 48 h of intranasal inoculation in BALB/c mice: olfactory mucosa (OM), olfactory bulb (OB), brain, trigeminal nerve (Tg) and lungs of mice inoculated with both a high (1 x 10^6^ IFU; N = 9 mice) and a low (1 x 10^3^ IFU; N = 9 mice) dose of *C. muridarum*. No *C. muridarum* bacteria were isolated from the tissues of control (vehicle only; N = 3) mice. The *C. muridarum* load from the OM was significantly different between mice inoculated with a high dose and a low dose (*p ≤ 0.05, Student**’**s T-test). Data are mean number of inclusions +/- SEM. **(B–N)** Example images of *C. muridarum* inclusions in sagittal tissue sections (immunolabelled for MOMP, green) with nuclei in blue (DAPI stain), rostral is to the left, dorsal to the top. Images are representative from five animals for each of two groups of mice inoculated with *C. muridarum* (high and low dose) and three control mice. Panels show maximum projection of confocal microscopy z-stacks. **(B)** Low power image showing the location of the nasal cavity (NC), olfactory epithelium (OE), OB, brain, and Tg (dotted line), yellow letters indicate approximate locations of panels **(C–N)**. Panels **(C–N)** show *C. muridarum* inclusions (green) in low dose and high dose inoculated mice in **(C, D)** the olfactory epithelium; **(E, F)** olfactory nerve fascicles (only low dose shown); panel **(F)** shows high power view of **(E)**; **(G, H)** the OB. **(I–L)** In the dorsal-caudal OB, discrete *C. muridarum* inclusions were detected; arrow in I shows location, **(J, K)** show two examples of *C. muridarum* inclusions, **(L)** control non-inoculated in dorsal-caudal OB. **(M, N)** *C. muridarum* inclusions in the trigeminal nerve. Scale bars in µm.

To determine whether *C. muridarum* could infect the primary olfactory nervous system and potentially progress to the CNS, we inoculated mice (BALB/c) with *C. muridarum* intranasally and analyzed tissue lysates and sections for the presence of the bacteria. The mice were inoculated with a low dose (1 x 10^3^ IFU) or a high dose (1 x 10^6^ IFU) of *C. muridarum*, or with vehicle alone. At 48 h post inoculation, we harvested the olfactory mucosa (which the olfactory epithelium and underlying lamina propria with olfactory nerves), olfactory bulb, and brain (beyond the olfactory bulb) and determined whether infectious *C. muridarum* bacteria could be isolated from the tissues. In addition, we analyzed the trigeminal nerve, since this nerve has previously been shown to be subject to invasion by other bacterial species (St John et al., 2016) and the lungs, which are readily infected by *C. muridarum* after intranasal inoculation in mice (thus serving as a positive control for infection) (Skelding et al., 2006). To determine the number of *C. muridarum* IFUs isolated from tissues, the tissue lysates were serially diluted onto McCoy B cells, a mouse fibroblast cell line highly susceptible to *C. muridarum* infection (Lyons et al., 2005). Twenty-four hours later, immunolabelling for *C. muridarum* (anti-MOMP) was performed and the number of inclusions were counted. Infectious *C. muridarum* organisms were isolated from all the tissues of animals inoculated with both the high and the low dose of *C. muridarum* (but not from control animals) (**Figure 2A**). Significantly more *C. muridarum* IFUs were isolated from the olfactory mucosa of mice inoculated with the high dose of *C. muridarum* than from mice that received the low dose. For the other organs there was no difference in bacterial load between high dose- and low dose-inoculated animals.

To confirm that *C. muridarum* invaded the olfactory mucosa, olfactory bulb, brain, and trigeminal nerve, we immunolabelled tissue sections of mouse heads for *C. muridarum* followed by confocal microscopy. Since *C. muridarum* EBs and RBs are less than 0.35 µm and 2 µm in diameter, respectively, detection of EBs and RBs microscopically is difficult. In contrast, the much larger inclusions can easily be detected. *C. muridarum* inclusions were detected within the olfactory epithelium (**Figures 2A, C, D**), olfactory nerve (**Figures 2E, F**) and the glomerular layer of the ventral olfactory bulb (OB) (**Figures 2G, H**) of all both low dose- and high dose-inoculated mice. Inclusions were typically found near nuclei. The irregular structure of some inclusions within the glomerular layer of the OB (**Figures 2G, H**) appeared similar to that previously shown to be indicative of aberrant bodies (Beatty et al., 1994). In some mice, *C. muridarum* inclusions were present in a discrete region of the dorsal-caudal OB (see **Figures 2B, I** for location; this occurred in two out of five mice for both low dose- and high-dose inoculated mice). These inclusions were isolated and located close to nuclei (**Figures 2J, K**); again, these inclusions appeared to be indicative of aberrant bodies. Immunolabelling of the dorsal-caudal OB in sections from control mice showed no immunoreactivity for *C. muridarum* MOMP (**Figure 2L**). *C. muridarum* inclusions were also found in the trigeminal nerve (in three out of five low dose-inoculated mice and in one out of five high dose-inoculated mice) (**Figures 2M, N**). Anti-*C. muridarum* immunolabelling of the olfactory epithelium, OB and trigeminal nerve of control mouse tissue is shown in **Supplementary Figure 1**. The brain was also examined for presence of *C. muridarum*, particularly in regions associated with the olfactory bulb and brainstem. However, despite infectious *C. muridarum* organisms being isolated from the brain beyond the OB, *C. muridarum* inclusions were not microscopically detected in these brain regions.

The localisation of *C. muridarum* inclusions in the olfactory epithelium, olfactory nerve and olfactory bulb suggests a direct route of entry from the nasal cavity to the olfactory bulb. However, to determine whether the bacteria could also potentially have reached the CNS *via* blood-borne macrophages (and/or infected olfactory nerve tissue *via* blood-to-nerve transmission), we also examined blood of inoculated mice using PCR. However, PCR analysis did not detect *C. muridarum* in the blood at 48 h post infection; this time was chosen for analysis as it would allow for one round of replication (**Supplementary Figure 2**).

We have previously developed a transgenic reporter mouse line, the S100β-DsRed line. In this strain, all glial cells express the red fluorescent protein DsRed driven by the S100β promoter, allowing microscopic visualization of glial cells, including olfactory ensheathing cells (OECs), Schwann cells (including TgSCs), and astrocytes (Windus et al., 2007). This mouse line also enables clear identification of olfactory nerve fascicles and the nerve fibre layer (NFL) of the OB (structures which are both populated by OECs) and thus, determination of whether bacteria progress from the olfactory epithelium to the OB *via* nerve fascicles. We therefore also inoculated S100β-DsRed mice intranasally with *C. muridarum* (here, we only used one dose, 1 x 10^6^ IFU/mouse) and analyzed tissue sections of the primary olfactory nervous system of these mice.

**Figure 3:**
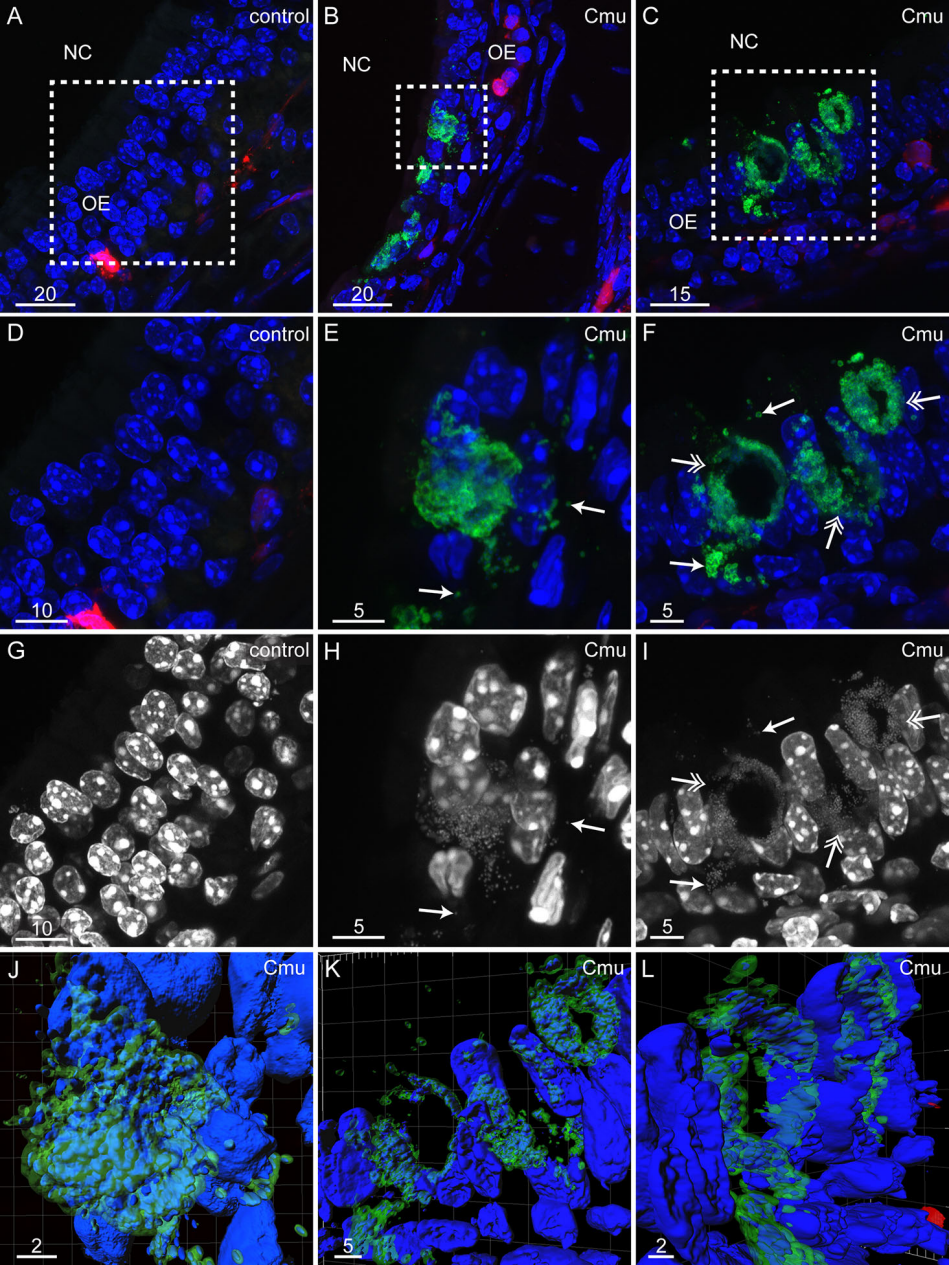
*C. muridarum* can invade the olfactory epithelium and form inclusions in S100β-DsRed mice 48 h post inoculation. Panels show coronal sections (maximum projections of z-stack images), from S100β-DsRed mice immunolabeled for *C. muridarum* (Cmu, green) with nuclei stained with DAPI (blue). Red: glial cells expressing the DsRed protein. **(A–C)** Low power images of the nasal cavity (NC) showing the olfactory epithelium (OE). **(A)** No immunofluorescence for *C. muridarum* was detected in control mice (vehicle only); a zoomed image of the area indicated by the white box is shown in panel **(D)**. **(B)** Clusters of *C. muridarum* inclusions were found within the OE (green); a zoomed image of the white box area shown in panel **(E)**. **(C)** Occasionally immunostaining for *C. muridarum* inclusions (green) was pronounced around the exterior of the inclusion (double-headed arrows). **(D)** A zoom of panel A (the OE of a control mouse) showing no immunolabelling for *C. muridarum*. **(E)** A zoomed image of panel B (the OE of a *C. muridarum*-infected mouse). A large *C. muridarum* inclusion (green) is surrounded by smaller immunoreactive particles which are also labelled with DAPI (arrows point to examples). **(F)** A zoomed image of panel C (inclusions with hollow centres in the OE of a *C. muridarum*–infected mouse). Double-headed arrows indicates inclusions (green) with hollow centres. Single-headed arrows indicates smaller particles immunoreactive to *C. muridarum* MOMP which are also stained by DAPI. **(G–I)** Images showing only the DAPI staining (blue) of images in panels **(D–F)**. Arrows shown in panels **(E, F)** are in the same position as those in panels **(H, I)**. **(J)** A 3D reconstruction of the *C. muridarum* inclusion in panels **(E, H)**. **(K, L)** 3D reconstructions of the hollow *C. muridarum* inclusions in panels **(F**, **I)** Images are representative of three control (vehicle only) mice and five *C. muridarum*-inoculated mice. Fifteen to 21 tissues sections per control mouse and 27–36 tissue sections from *C. muridarum*-inoculated mice were screened. Scale bars in µm.

**Figure 4:**
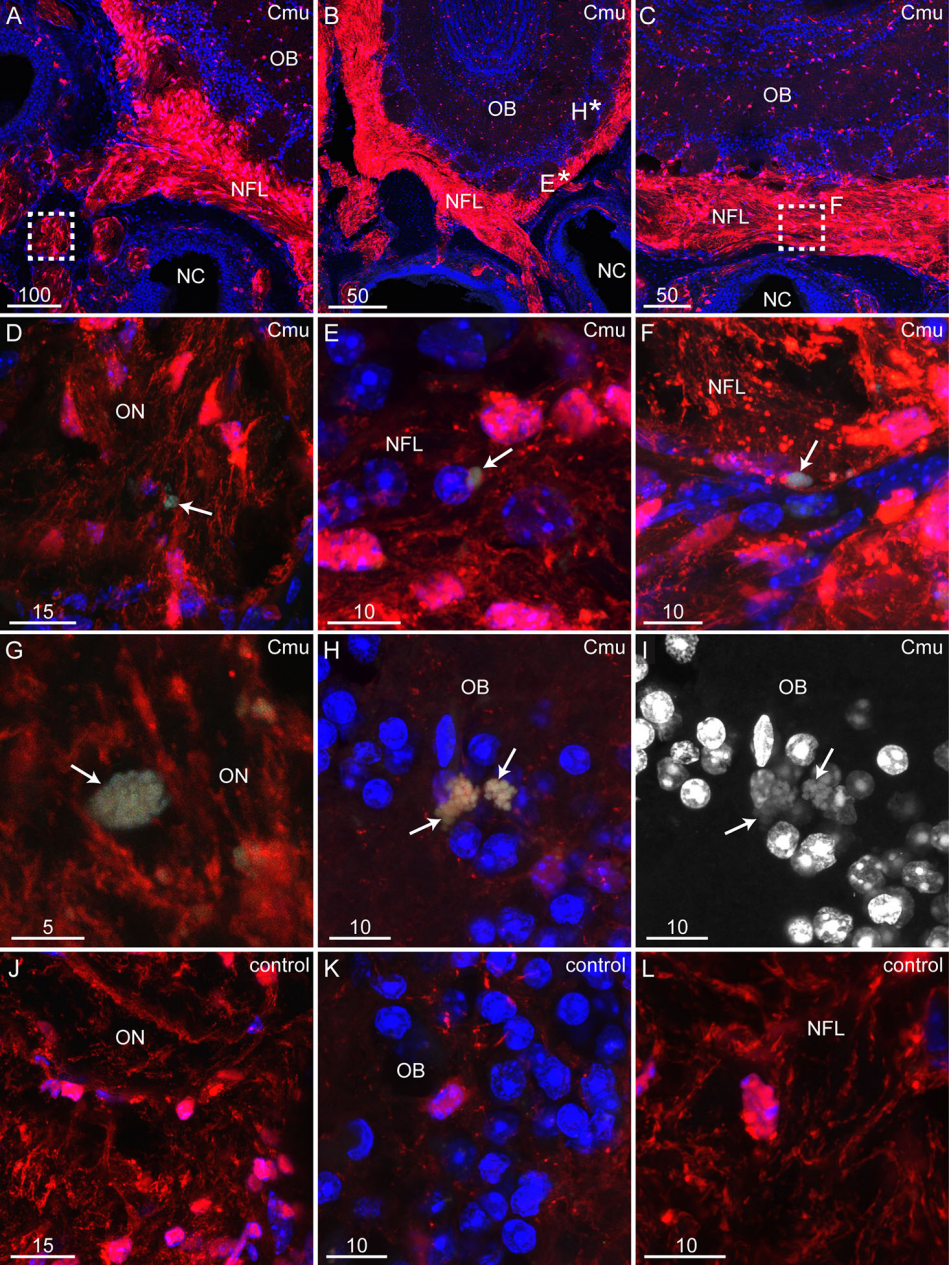
*C. muridarum* invades the olfactory nerve and bulb in S100β-DsRed mice. Panels show coronal sections from S100β-DsRed mice immunolabeled for *C. muridarum* (green) with nuclei stained with DAPI (blue). Red: glial cells expressing the DsRed protein. All images are maximum projections of z-stacks. **(A–C)** Low power images showing the coronal anatomy of the olfactory bulb (OB) in relation to the nasal cavity (NC). **(A)** Image showing the NC, nerve fibre layer (NFL) and OB. Region within the white box is the olfactory nerve (ON, containing DsRed-expressing OECs) and is zoomed in panel **(D)**. **(B)** The regions indicated by * are shown in more detail in different panels. The NFL, E*, is shown in panel E while the OB, H*, is shown in panel **(H)**. **(C)** The region of the NFL indicated by the white box is zoomed in panel **(F)**. **(D)** A zoomed image of the olfactory nerve (ON; red) shown by the white box in panel **(A)**. A *C. muridarum* inclusion within the olfactory nerve (green; arrow). **(E)** A zoomed image of the NFL indicated by the E* in panel **(B)**. Arrow points to a *C. muridarum* inclusion (green) within the NFL. **(F)** A zoomed image of the NFL indicated by the white box in panel **(C)**. Arrow points to a *C. muridarum* inclusion (green). **(G)** A high magnification image of a *C. muridarum* inclusion (green; arrow) within the olfactory nerve (red; ON). **(H)** High magnification image showing two *C. muridarum* inclusions (green; arrows) within the OB (red). Location of image indicated by H* in panel **(B)**. **(I)** Same image as shown in panel H with only the DAPI channel visible. Arrows point to the same inclusions as indicated in panel **(H)**. **(J–L)** Images of tissue sections from mice inoculated with vehicle only (HBSS). No immunolabelling for *C. muridarum* was detected within the ON **(J)** or OB **(K)**, including within the NFL **(L)**. Images are representative of three control mice and five infected mice. Scale bars in µm.

As for BALB/c mice, *C. muridarum* inclusions were detected throughout the primary olfactory nervous system of S100β-DsRed mice (**Figures 3** and **4**; for these experiments, we analyzed three control (vehicle only) mice and five *C. muridarum*-inoculated mice). In the olfactory epithelium, *C. muridarum* inclusions were often observed in clusters (**Figures 3A–C**). Higher magnification imaging revealed the presence of smaller particles immunoreactive for MOMP (**Figures 3D–F**). Occasionally, in *C. muridarum* inclusions, the anti-MOMP immunoreactivity was pronounced around the exterior of the inclusions with little immunolabelling in the centre (**Figure 3F**). While we observed this infrequently, it was detected in tissue sections in which other inclusions displayed immunoreactivity throughout the inclusion and it was not observed in smaller inclusions, so it was not an artefact of the fixation or preparation method. The DNA of *C. muridarum* (DAPI stain) could also be detected and differentiated from endogenous nuclei in the epithelial tissue (**Figures 3G–I**). 3D reconstructions of the high-resolution images further confirmed the presence of bacterial DNA within the inclusions expressing MOMP (**Figures 3J–L**).

We found that *C. muridarum* inclusions were present within olfactory nerve fascicles in two out of the five *C. muridarum*-inoculated S100β-DsRed mice (**Figures 4A, D, G**). We also detected *C. muridarum* inclusions in the NFL of the olfactory bulb in all of the inoculated mice (**Figures 4B, C, E, F**) and in the glomerular layer of four out of five inoculated mice (**Figures 4H, I**). Similar to the inclusions seen in the glomerular layer of the OB in BALB/c mice, *C. muridarum* inclusions within the OB of S100β-DsRed mice were often not uniformly round but had an elongated structure similar to aberrant bodies (**Figure 4I**) (Beatty et al., 1994). No MOMP immunolabelling was detected in control mice (**Figures 4J–L**).

### *Chlamydia muridarum* Infects Cultured Glial Cells From the Olfactory and Trigeminal Nerves

Since we found that *C. muridarum* infected the olfactory and trigeminal nerves *in vivo*, we next determined how OECs and TgSCs responded to the bacteria *in vitro*. We also included McCoy B cells (a mouse fibroblast cell line) as a positive control as these cells are readily infected with *C. muridarum* and routinely used to propagate the bacteria. OECs and TgSCs (peripheral nerve glia) are not considered “professional phagocytes” like macrophages/microglia (Jung and Chung, 2018). Macrophages can become infected by *Chlamydia* species, but can also respond to and reduce intracellular survival of the bacteria, in particular polarized M1 macrophages (Gracey et al., 2013; Herweg and Rudel, 2016; Islam et al., 2019; Rajaram and Nelson, 2015). To compare the OEC/TgSC responses to *C. muridarum* with that of professional phagocytes, we also included unstimulated macrophages representative of the M0 state (the J774A.1 mouse macrophage cell line). Unstimulated macrophages were used as neither the OECs nor Schwann cells were stimulated prior to addition of the bacteria.

**Figure 5:**
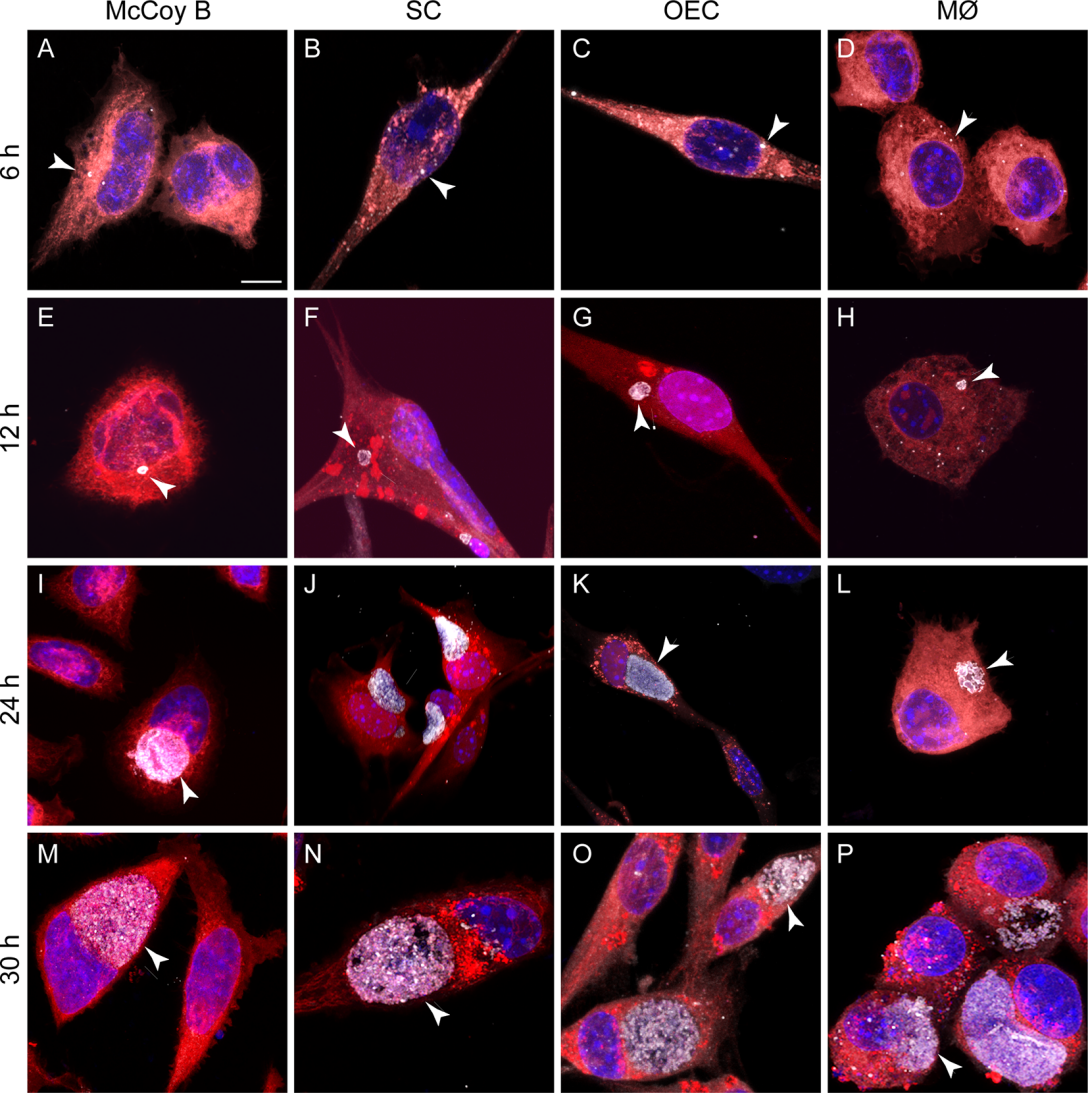
*C*. *muridarum* infection of cultured peripheral glia, macrophages and McCoy B cells. Shown are confocal images of McCoy B cells, TgSCs, OECs and J774A.1 macrophages, infected with *C. muridarum* (MOI 2:1). TgSCs and OECs express DsRed; McCoy B and J774A.1 are stained with cell mask (orange). *C. muridarum* inclusions (arrows) are labelled with anti-MOMP (white). Blue: nuclear stain (Hoechst). Cells were fixed and imaged at the times indicated at the left side of the panels. **(A–D)** 6 h, **(E–H)** 12 h, **(I–L)** 24 h, and **(M–P)** 30 h post infection. Scale bar: 10 µm.

We isolated primary OECs and TgSCs from S100β-DsRed mice (Windus et al., 2007; Panni et al., 2013) and inoculated the cultured glia, along with McCoy B cells and J774A.1 macrophages, with *C. muridarum* at different MOIs. The medium was changed after four h, and cells were analyzed for up to post exposure to the bacteria. Immunoreactivity for *C. muridarum* (anti-MOMP) was detected in all four cell types from 6 h onwards, with large inclusions present close to nucleus and the microtubule organising centre from 24 h onwards (**Figure 5**).

**Figure 6:**
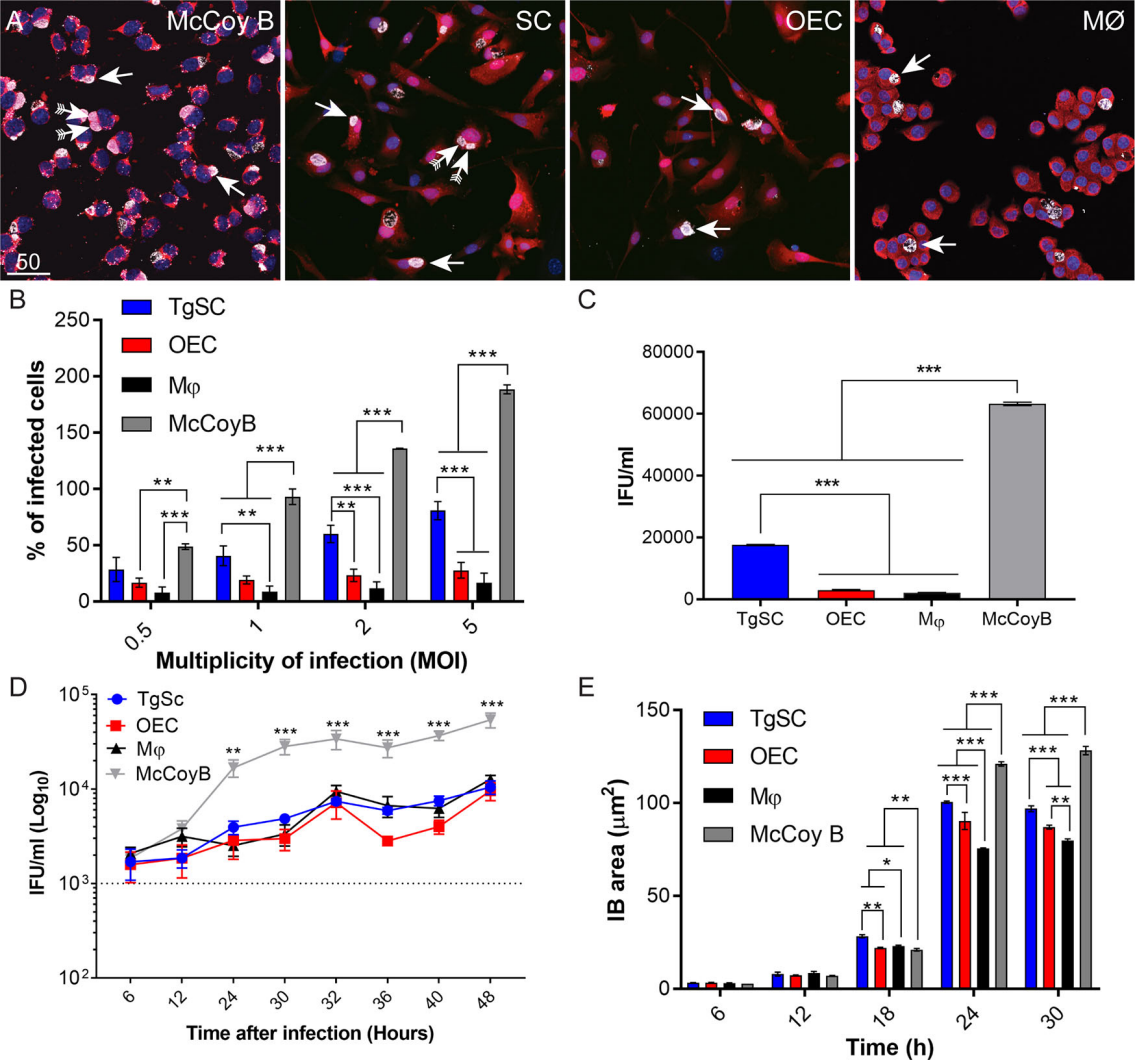
Comparison of characteristics of *C. muridarum* infection of peripheral glia, macrophages and McCoy B cells after 24 h. **(A)** Confocal images of McCoy B cells, TgSCs, OECs, and macrophages, respectively, infected with *C. muridarum* at MOI of 2:1. TgSCs and OECs express DsRed, while McCoy B and J774A.1 macrophages are stained with cell mask (orange). *C. muridarum* inclusions (arrows) are labelled with anti-MOMP (white); blue: nuclear stain (Hoechst). Most infected cells had one inclusion, however some had more than one (arrows with tails). Scale bar: 50 μm. **(B)** Percentages of infected cells containing *C. muridarum* inclusions 24 h post inoculation (p.i.) at different MOIs. **(C)** Viability assay. Cells were lysed at 24 h p.i (MOI 2:1) followed by inoculation of a McCoy B cell monolayer and IFU count. **(D)** Growth curves. All cell types were inoculated with *C. muridarum* (MOI 0.5:1) and lysed at different time-points p.i. (X-axis). The lysates were then used to inoculate a monolayer of McCoy B cells, and the number of IFUs/ml of lysate was determined (Y-axis, logarthmic scale). The limit of detection for the assay indicated as a dotted line. **(E)** Inclusion area size. For all these graphs, data represent mean +/- SEM and n = 3 biological repeats x 3 technical replicates x 4,000 cells. *P ≤ 0.05, **P ≤ 0.01, ***P ≤ 0.001. Growth Kinetics, % infection and inclusion size: two-way ANOVA and Tukey’s post-hoc test. Re-infection 24 h: Ordinary one-way ANOVA with Tukey’s multiple comparison test.

To determine if there was any difference in the ability for *C. muridarum* to infect and form inclusions in the different cell types, we then inoculated the cells at different MOIs and determined the percentage of infected cells at 24 h post inoculation. At lower MOIs, a single inclusion was typically observed per cell, however at higher MOIs, multiple inclusions were sometimes observed (**Figure 6A**). Hence, percentage infection was determined by dividing number of IFUs per well with the initial host cell seeding density. The percentage of infected cells was higher for McCoy cells than for TgSCs, OECs, and macrophages (**Figure 6B**). There was also a difference in the percentage of infected cells between the two glial cell types. At MOIs of 2:1 and 5:1, TgSCs had a significantly higher infection incidence than OECs. At MOIs 1:1, 2:1, and 5:1, the number of infected TgSCs was also higher than the number of infected macrophages. We also recorded cells numbers based on nuclear count (Hoechst). At MOI 5:1 there was significant reduction in cell numbers as compared to the control, showing that *C. muridarum* at this MOI caused cell death (data not shown). Thus, for the following assays we used a MOI of 2:1, with the exception of growth curve assay for which a MOI of 0.5:1 was used.

### *Chlamydia muridarum* Can Survive and Replicate Inside Glial Cells

*C. muridarum*, like other *Chlamydia* species, has a biphasic lifecycle. Infective EBs enter the host cell, become RBs inside the inclusion bodies, and then revert back to EBs that can in turn infect other cells (see **Figure 1**) (Lyons et al., 2005). The fact that typical *C. muridarum* inclusions were detected in cells using immunocytochemistry suggests that the bacteria were not simply phagocytosed by the host cell but instead survived and replicated intracellularly. To verify that the bacteria were viable and could generate infectious progeny, however, isolation of infectious EBs from the host cells, which could then in turn infect new cells, was necessary. Infected host cells were lysed 24 h post exposure to bacteria (hereafter referred to as post inoculation; p.i.), and the lysates were used to inoculate a monolayer of McCoy cells. This time point was chosen to capture the EBs prior to their natural lysis (or extrusion) from the cells, which occurs from 24–30 h p.i. The number of resultant inclusions were counted; the number of inclusions constitutes the number of viable IFUs obtained from each cell type. Similar to our infection studies, the amount of IFUs isolated was higher for McCoy B cells than the other cell types. A significantly higher number of IFUs were also isolated from TgSCs than from OECs and macrophages (**Figure 6C**).

The time-course of the *C. muridarum* life-cycle is thought to be variable between host cell types. Although the life-cycle of *C. muridarum* has been well established in McCoy B cells (24–36 h) (Carey et al., 2013; Huang et al., 2015), the growth kinetics in glial cells remains unknown. Whilst it has been assumed that *C. muridarum* has an extended life-cycle of ~48 h in macrophages (Lyons et al., 2005; Gracey et al., 2013), the life-cycle in these cells still remains somewhat elusive. Therefore, our next aim was to address potential variation of *C. muridarum* growth kinetics in glial cells, in comparison to macrophages and McCoy B cells. To determine the lifecycle of *C. muridarum* in the different cell types, a growth curve assay was conducted. For these experiments, host cells were lysed at different time-points p.i. (6, 12, 24, 30, 32, 36, 40, and 48 h p.i.), followed by inoculation of McCoy B cells as described above. Since this study requires culture over a longer time (48 h), a MOI of 0.5:1 was used to prevent over-infection of the host cells.

Between 6–12 h p.i., a negligible amount of infectious progeny (IFUs) was recovered from all the cell types (**Figure 6D**; shown as IFUs/mL of cell lysate). IFUs were first detected from McCoy B cells at 24 h p.i, the yield of which steadily increased over time and peaked at 32 h p.i. There was a decline in the amount of IFUs isolated between 32–36 h, indicating the commencement of the second life-cycle and potential conversion of EBs into RBs in the newly infected cells. There was no difference in growth kinetics (time-course of the life-cycle) between the different cell types. The yield of IFUs, however, was significantly higher from McCoy B cells than from the other cell types from 24 h onwards. This suggests that growth of *C. muridarum* is less favourable in glia and macrophages than in McCoy B cells.

To determine whether the inclusion size correlated with yield of infectious organisms in the different cell types, we also analyzed the size of inclusions within these cells over time. At early time-points (less than 24 h p.i.), no differences in inclusion size between cell types was observed. However, at later times (24 and 30 h p.i.) the mean inclusion size was significantly smaller in glia and macrophages than in McCoy B cells (**Figure 6E**). We also measured the area of the different cell types to investigate if the area of the IB was influenced by the size of the host cell. OECs and Schwann cells were the largest cells with an area of 1,251 µm^2^ ± 27.2 and 1,169 µm^2^ ± 44.7 respectively followed by McCoy B (931 µm^2^ ± 38.7) and then macrophages (694.38 µm^2^ ± 15.27). Statistical analysis one way Anova with tukey’s multiple comparison test revealed that OECs and Schwann cell size were not significantly different, but that OECs were significantly larger than McCoy B (p < 0.0001) and macrophages (p < 0.0001), and Schwann cells were significantly larger than McCoy B cells (p < 0.001) and macrophages (p < 0.0001). Small inclusion size has been correlated to restricted growth in dendritic cells and macrophages (Rey-Ladino et al., 2007; Kaiko et al., 2008; Gracey et al., 2013). While glia have a larger area than McCoy B cells, they contained significantly smaller IBs (**Figure 6E**) indicating that OECs and Schwann cells might not contain the most hospitable environment required for the development of the bacteria.

### *Chlamydia muridarum* Infection of Glia Results in the Production of Cytokines, With OECs Exhibiting a Stronger Cytokine Response Than TgSCs

**Figure 7:**
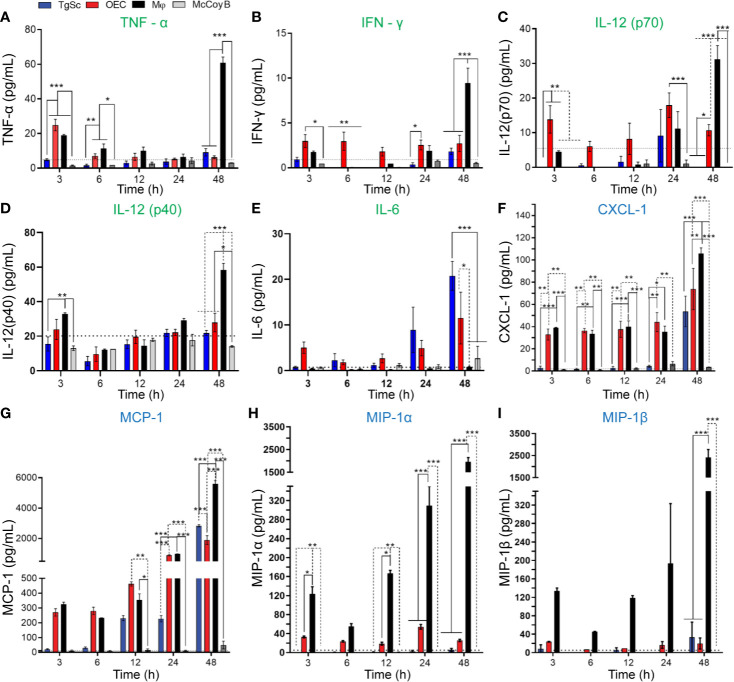
Cytokine and chemokine secretion by OECs, TgSCs, macrophages, and McCoy B cells following *C. muridarum* infection. Cells were inoculated at MOI 2:1. After 3, 6, 12, 24, and 48 h post infection, supernatants were collected and assayed using a Bioplex-23-plex kit. The different cell types, in particular, OECs and macrophages, produced a range of pro-inflammatory cytokines, **(A)** IFN-γ, **(B)** TNF-α, **(C)** IL-12(P70), and **(D)** IL-12(P40). OECs and TgSCs also produced the regulatory cytokine **(E)** IL-6 while chemokines **(F)** CXCL-1, **(G)** MCP-1, **(H)** MIP-1α, and **(I)** MIP-1β were produced by TgSC, OECs, and macrophages. Cells that had not been infected (control cells) did not produce cytokines/chemokines at detectable levels. Data are shown as mean ± SEM; n = 3 independent experiments. *p ≤ 0.05, **p ≤ 0.01, ***P ≤ 0.0001, two-way ANOVA with Tukey’s post-hoc test.

To determine whether OECs and TgSCs responded to *C. muridarum* infection by secretion of key cytokines and chemotactic cytokines (chemokines), the cells were infected with *C. muridarum* (MOI 2:1) and the cytokine/chemokine production was assessed at different time-points (3, 6, 12, 24, and 48 h p.i.). Production of these cytokines was also assessed for macrophages and McCoy B cells at the same time-points for comparison. *C. muridarum* infection resulted in increased secretion of pro-inflammatory cytokines interferon γ (IFN-γ), tumour necrosis factor α (TNF-α) and interleukin 12 (both IL-12p40 and IL-12p70, out of which the heterodimeric IL12p70 is the bioactive form) by OECs and macrophages (**Figure 7**). Production of the regulatory cytokine interleukin 6 (IL-6) was detected for the glial cells (both OECs and TgSCs), but not macrophages, at all time-points p.i. For IFN-γ, only OECs responded to infection with increased levels at time-points prior to 48 h p.i, whereas at 48 h p.i., macrophages also secreted high levels of this cytokine; at this time-point, macrophages produced higher levels of IFN-γ than OECs (**Figure 7B**). Both OECs and macrophages secreted TNF-α soon after exposure to *C. muridarum*. At 48 h p.i., however, macrophages strongly increased production of TNF-α whilst levels produced by OECs at this time-point returned to baseline (**Figure 7B**). OECs and macrophages secreted higher levels of IL-12p70 than the other cell types at all time-points, with macrophages producing higher levels than OECs (**Figure 7C**). Infection also led to the secretion of the chemokines C-X-C motif ligand 1 (CXCL-1, also known as KC or Gro), monocyte chemoattractant protein 1 (MCP-1), macrophage inflammatory proteins (MIP-1α and MIP-1β, also known as CCL3 and CCL4) by OECs and macrophages. TgSCs secreted only MIP-1α (at all time-points p.i.) and MCP-1 at 48 h p.i in response to infection (**Figures 7F–I**). All data for the cytokine/chemokine multiplex assays (the full panel of cytokines/chemokines assessed and amounts in pg/ml) are shown in **Supplementary Table I**.

## Discussion

We have demonstrated that *Chlamydia muridarum* can invade the primary olfactory nervous system (olfactory nerve and bulb) and the trigeminal nerve within 48 h of intranasal inoculation in mice. We showed that infectious *C. muridarum* organisms could be isolated from these tissues, and we detected *C. muridarum* inclusions in histological sections of these anatomical regions. We also isolated viable *C. muridarum* from the cerebral cortex but, despite screening numerous tissue sections, did not detect any evidence of *C. muridarum* immunolabelling in the cerebral cortex. This suggests that *C. muridarum* inclusions in the cortex are sparse or, at this time-point post infection, very small and difficult to detect.

The results presented in the current study are the first to show that *C. muridarum* can invade the olfactory epithelium and progress into the olfactory bulb (OB) *via* olfactory nerve fascicles. Invasion of the OB at 48 h p.i. is considerably earlier than what has previously been shown for *C. pneumoniae* infection in mice (7-14 days) (Little et al., 2005; Boelen et al., 2007). We did not immunohistologically detect spread of *C. muridarum* into deeper regions of the brain within the infection time of this analysis. Longer term studies may reveal this, but the potential spread is also likely to be dependent on genetic susceptibility, which can be tested with various genetic mouse models. As an example with another bacterium, we have previously demonstrated that *B. pseudomallei* can readily penetrate the trigeminal nerve of S100β-DsRed transgenic mice (St John et al., 2016) but not BALB/c mice (St John et al., 2014), indicating that genomic differences drive susceptibility to particular nerve pathways.

Whilst infection of the OB by *C. pneumoniae* has been presumed to be *via* the olfactory nerve and/or *via* blood, the presence of these bacteria in olfactory nerve fascicles has not previously been shown. *C. muridarum* was not detected in blood by PCR, suggesting the bacteria reached the OB *via* entry from the olfactory epithelium and nerve. Infection of the trigeminal nerve by *C. muridarum* shown here also constitutes the first evidence of Chlamydial infection of this nerve.

Within the OB, *C. muridarum* was mainly localized within the ventral bulb. However the bacteria was also localized in a discrete region in the dorsal-caudal OB in some mice, without detectable inclusions being present in the region between the ventral bulb and dorsal-caudal bulb. The reason for this disparate localization is unknown, but potentially could be related to the differential expression of carbohydrates by primary olfactory axons that contribute to the complex olfactory topographical map (St John and Key, 2001).

We inoculated mice with both a high and a low dose of *C. muridarum* (1x10^3^ and 1x10^6^ IFU, respectively), the lower dose being the same as the dose readily infecting the lungs after intranasal *C. muridarum* infection (Horvat et al., 2007; O’Meara et al., 2013). A significantly higher number of IFUs were isolated from the olfactory epithelium of high dose- than low dose-inoculated mice. However, a similar number of IFUs were isolated from all other tissues tested (trigeminal nerve, OB, the rest of the brain and the lungs) from low dose- and high dose-inoculated animals. This suggests that even a low dose of *C. muridarum* can rapidly ascend to the CNS after intranasal inoculation. A previous study assessing *C. muridarum* invasion of the lower and upper genital tract in mice showed that a low inoculation dose resulted in the recovery of more viable organisms from the oviducts than a high dose (Maxion et al., 2004).

Since *C. muridarum* is an obligate intracellular bacterium with a lifecycle of ~ 32–56 h (Ramsey et al., 2009; Bryan et al., 2019), the fact that *C. muridarum* invaded sites distant from the nasal epithelium so rapidly suggests that chlamydial EBs may rapidly “travel” (possibly by flow of extracellular fluid) *via* nerve fascicles into the CNS. It is also possible that localized *C. muridarum* infection in the nasal epithelium causes damage to the olfactory mucosa, allowing access to the underlying nerve fascicles. Infection-induced injury may also cause death of some olfactory neurons, allowing *C. muridarum* to access the olfactory bulb *via* empty olfactory nerve fascicles. This appears to be how *B. pseudomallei* invades the olfactory bulb (St John et al., 2014). *B. pseudomallei*, however, is a rod-shaped motile facultative intracellular bacterium and thus strikingly different from *Chlamydia* species.

The glial cells of the olfactory nerve (OECs) are hypothesized to be the key phagocytes in this nerve, and to play key roles in the protection against microorganisms (Harris et al., 2009; Panni et al., 2013; Nazareth et al., 2015). The ability to infect and survive inside OECs, rather than being phagocytosed and degraded by the cells, is thought to be a feature of microorganisms that can invade the primary olfactory nervous system (Macedo-Ramos et al., 2016; Walkden et al., 2020). Trigeminal nerve Schwann cells (TgSCs) can also phagocytose microorganisms (Panni et al., 2013), however, Schwann cells have also been shown to express lower levels of mRNA for innate immune factors (Vincent et al., 2005) and to mount a far weaker immune response to bacteria (Vincent et al., 2007) than OECs. In these previous studies, the Schwann cells were isolated from spinal nerves and not the trigeminal nerve; due to their location, we hypothesize that TgSCs may be more often exposed to bacteria than Schwann cells elsewhere and may therefore have evolved to better withstand pathogen insult.

In the current study, we demonstrated that both OECs and TgSCs could be infected by *C. muridarum*, and that infectious progeny could be isolated from these cells over time. The capacity for infecting and surviving in glia, rather than being degraded, may thus be one reason for which *C. muridarum* can invade the primary olfactory nervous system and trigeminal nerve. We showed that the bacterial yield and percentages of infected cells was significantly lower in glia than in McCoy B fibroblasts, the cell line typically used to propagate *C. muridarum*. We also showed that Chlamydial growth was less restricted in glia than in macrophages. Thus, *C. muridarum* has less capacity for growth in glia than in McCoy B cells, but the glia do not have as strong bactericidal properties as macrophages. For some conditions, OECs appeared to better withstand Chlamydial infection than TgSCs, potentially suggesting that OECs exhibit stronger immune responses than TgSCs.

Whilst the yield was lower in glia than in McCoy B cells, a relatively large number of infectious organisms were still isolated from the glia (even at low MOIs). The ability to rapidly generate large numbers of EBs may contribute to the rapid progression through the primary olfactory nervous system, trigeminal nerve and CNS. The capacity for fast production of progeny has been suggested to be a key reason for why *C. muridarum* can rapidly colonize the lower and upper genital tracts (Lyons et al., 2005). We also showed that for all cell types tested, the life-cycle of *C. muridarum* was ~32 h, which is similar to what has previously been shown for McCoy B cells (Skilton et al., 2018).

We also showed that *C. muridarum* infection resulted in the production of pro-inflammatory cytokines (IFN-γ, TNF-α, IL-6 and IL-12), and chemokines (CXCL-1, MCP-1, MIP-1α/β) by the cultured glia and macrophages. Overall, OECs and macrophages produced significantly higher levels of these factors than TgSCs. Together, these data show important differences between OECs and TgSCs in terms of innate immune responses to *C. muridarum*, with OECs but not TgSCs producing cytokines and chemokines at significant levels, and with TgSCs showing higher levels of infection (percentage of infected cells and bacterial yield for some conditions). These findings are in alignment with previous studies suggesting stronger immune responses by OECs than Schwann cells (Vincent et al., 2005; Vincent et al., 2007).

Production of IFN-γ, which promotes activation of innate and adaptive immunity in response to pathogens, by these glia has not been previously described. The fact that peripheral nerve glia can secrete IFN-γ in response to bacteria is somewhat surprising, since this cytokine is primarily considered a T-cell/NK-cell cytokine (Schoenborn and Wilson, 2007), however, IFN-γ has also been shown to be produced by human macrophages (Darwich et al., 2009).

Whilst not assessing secretion of IFN-γ in response to bacteria, one study has shown that treatment with IFN-γ can protect against *Streptococcus pneumoniae* infection of OECs (Macedo-Ramos et al., 2016). In the case of *Chlamydia* species, however, IFN-γ can induce the bacteria to enter persistence (Panzetta et al., 2018). Thus, the fact that OECs respond to *C. muridarum* infection with IFN-γ secretion may in the longer-term result in persistence.

TNF-α can attract immune cells to the area, which may aid in bacterial clearance, but also cause damage to neural tissues (Feuerstein et al., 1994). TNF-α has been shown to cause degradation of the olfactory epithelium and underlying neurons in mice (Lane et al., 2010), but also to be involved in regeneration after injury (Chen et al., 2017). Since the nasal epithelium and dendrites of primary olfactory neurons are exposed to the external environment, TNF-α activity in the primary olfactory nervous system is likely tightly regulated. For example, OECs can produce pituitary adenylate cyclase activating peptide (PACAP) (Hegg et al., 2003), which can protect against TNF-α-induced death of primary olfactory neurons (Kanekar et al., 2010).

IL-12 is mainly produced by multiple immune cells such as monocytes, macrophages, dendritic cells and B cells, and influences both the innate and adaptive immune response to infection (Trinchieri, 2003). Whilst not previously shown to be produced by TgSCs, the Schwann cells of the sciatic nerve produce bioactive IL-12 (Turka et al., 1995).

The active form of IL-12, IL-12p70, is a heterodimer formed by IL-12p40 and IL-12p35. IL-12p40 can also in itself act as an IL-12 antagonist (Chen et al., 2013). While IL-12p35 was not included in the panel, OECs and macrophages secreted both IL-12p40 and IL-12p70 with a similar time-course following *C. muridarum* infection.

IL-6 is a multifunctional cytokine known to have both pro- and anti-inflammatory effects (Scheller et al., 2011). IL-6 has previously been shown to be important for *C. muridarum* infection in mice, with mice lacking IL-6 being highly susceptible to airway infection by *C. muridarum* in comparison to wild type mice (Williams et al., 1998). In the olfactory nervous system, IL-6 likely has important roles with increased IL-6 production reported to occur throughout the primary olfactory nervous system and olfactory bulb after injury and infection (Herbert et al., 2012), with OECs shown to produce IL-6 in response to *S. aureus and B. pseudomallei* infection (Herbert et al., 2012; Dando et al., 2016).

Chemokines, including CXCL-1, MCP-1 and MIP-1 mediate recruitment of leukocytes and typically are produced secondary to pro-inflammatory cytokines (Graves and Jiang, 1995). In the nervous system, chemokines are involved in neural repair and neuroinflammation (Miller et al., 2008), however, their roles in the primary olfactory nervous system are unknown but likely involves attraction of immune cells in response to pathogen invasion. OECs, but not Schwann cells, have previously also been shown to secrete CXCL-1 in response to *E. coli* and PAMPs (Vincent et al., 2007). Both CXCL-1 and MCP-1 are also produced by both microglia and astrocytes in the CNS and are upregulated in the brain after trauma (reviewed by Semple et al., 2010) and microglia also express MIP-1 (Fang et al., 2011).

## Conclusion

In the current study, *C. muridarum* was shown to rapidly (within 48 h) invade the CNS *via* the olfactory nerve in mice after intranasal inoculation, even at a low inoculation dose. This study also provides the first evidence of a *Chlamydia* species infecting the trigeminal nerve. We also showed that *C. muridarum* infected the glial cells of both the olfactory and the trigeminal nerves, OECs and TgSCs, respectively. This may constitute a key reason for why these bacteria can invade the primary olfactory nervous system and trigeminal nerve. Furthermore, we showed that the glia responded differently to *C. muridarum.* OECs were less sensitive to infection and secreted significantly higher levels of cytokines and chemokines than TgSCs, corroborating previous findings showing that OECs mount a stronger immune response to pathogens than Schwann cells. Overall, these results demonstrate that while *C. muridarum* can infect the glia, the *C. muridarum* developmental cycle is delayed and the inclusion size is smaller than in McCoyB epithelial cells, indicating that the glia are not a favourable environment for intracellular *C. muridarum* growth. While the organ load data of the *in vivo* infections showed that *C. muridarum* was found within the brain, inclusions were not detected in the brain using immunohistochemistry. Together these results suggest that while *C. muridarum* can infect the olfactory and trigeminal nerves, the glia offer some degree of protection against dissemination into other regions of the nervous system.

## Data Availability Statement

The original contributions presented in the study are included in the article/**Supplementary Material**; further inquiries can be directed to the corresponding author.

## Ethics Statement

All procedures were approved by Griffith University’s Animal Ethics Committee (ESK/02/15/AEC and MSC/08/18/AEC) under the guidelines of the Australian Commonwealth Office of the Gene Technology Regulator.

## Author Contributions

LN, HW, AC, AD, JE, and JS planned and designed the experiments. Experiments were conducted by LN, HW, AC, AD, TS, CA, RR, and LT. HW, LN, AC, AD, JS, KB, and JE analyzed the data. HW and LN drafted the manuscript. All authors reviewed and edited the manuscript. JE and JS funded the research. JE provided the overall supervision of the project. All authors contributed to the article and approved the submitted version.

## Funding

This work was funded by a Menzies Health Institute Queensland grant to JE, a GODA Foundation grant to JE, and a Clem Jones Foundation Grant to JS and JE. HW and LN were supported by the Australian Government Research Training Program scholarships, and RR was supported by a Griffith University International Postgraduate Research Scholarship.

## Conflict of Interest

The authors declare that the research was conducted in the absence of any commercial or financial relationships that could be construed as a potential conflict of interest.
