# Supplementary material for: Chlamydia muridarum Can Invade the Central Nervous System via the Olfactory and Trigeminal Nerves and Infect Peripheral Nerve Glial Cells
Source: Front Cell Infect Microbiol. 2021 Jan 8;10:607779. doi: 10.3389/fcimb.2020.607779 (PMC7819965; doi:10.3389/fcimb.2020.607779)
Supplement: Supplementary file 1 [file DataSheet_1.docx]

*Chlamydia muridarum* can invade the central nervous system via the olfactory and trigeminal nerves and infect peripheral nerve glial cells.

Nazareth L, Walkden H, Chacko A, Delbaz A, Shelper T, Armitage C, Reshamwala R, Trim L, St John JA, Beagley KW, Ekberg JA

Supplementary Figure 1. Control tissue immunostaining for *C. muridarum.* Panels show confocal microscopy imaging of tissue sections of olfactory epithelium (OE), olfactory bulb (OB), and trigeminal nerve (Tg) from control non-inoculated mice stained with anti-MOMP antibodies (green); nuclei are stained with DAPI (blue). Scale bars in µm.

Supplementary Figure 2. Amplification curves; (+) shows positive control and (-) shows negative control. Based on the curves all blood samples were detected below the threshold suggesting absence of bacteria in blood one day post infection. Low dose n=9, high dose n=9, non-inoculated n=3.

Supplementary movie 1. Three-dimensional reconstruction of *C. muridarum* inclusion within cells in the dorsal olfactory bulb. Z projection of the confocal image is shown in Figure 2 K.
