# Supplementary figures and images for: Chlamydia muridarum Can Invade the Central Nervous System via the Olfactory and Trigeminal Nerves and Infect Peripheral Nerve Glial Cells
Source: Front Cell Infect Microbiol. 2021 Jan 8;10:607779. doi: 10.3389/fcimb.2020.607779 (PMC7819965; doi:10.3389/fcimb.2020.607779)

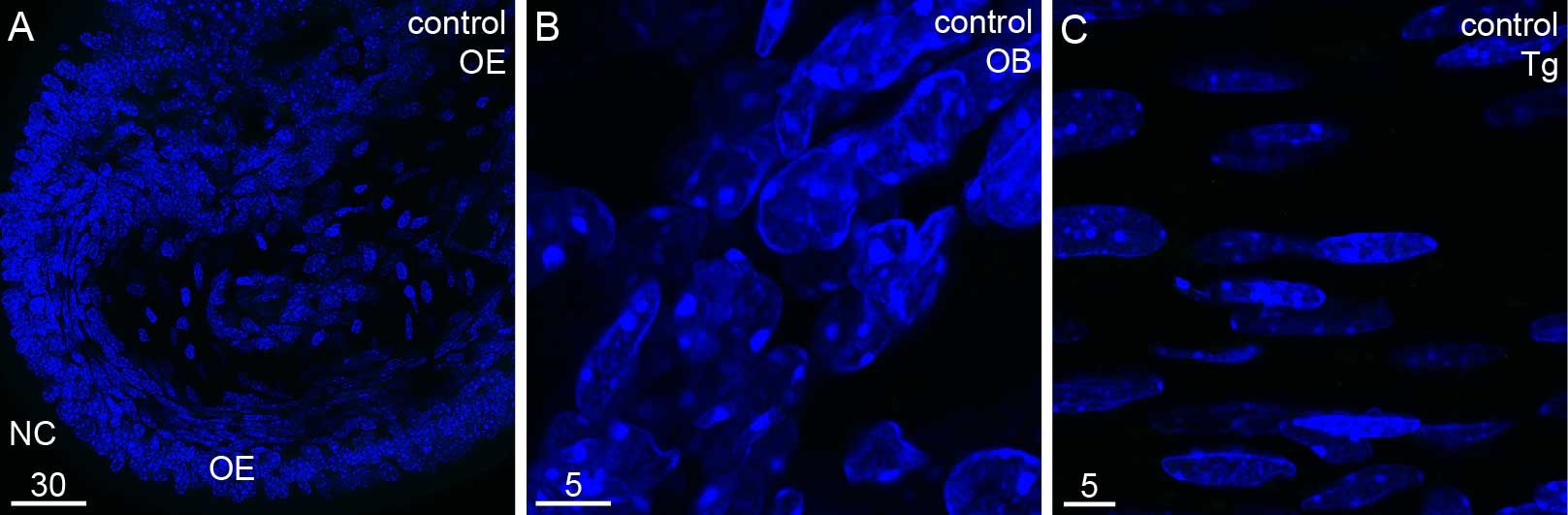

Supplement: Supplementary file 2 [file Image_1.jpg]

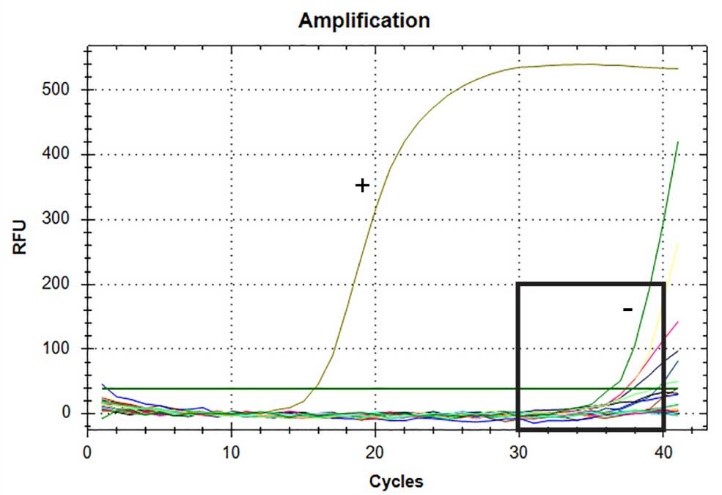

Supplement: Supplementary file 3 [file Image_2.jpg]
