# Supplementary material for: Chlamydia muridarum Can Invade the Central Nervous System via the Olfactory and Trigeminal Nerves and Infect Peripheral Nerve Glial Cells
Source: Front Cell Infect Microbiol. 2021 Jan 8;10:607779. doi: 10.3389/fcimb.2020.607779 (PMC7819965; doi:10.3389/fcimb.2020.607779)
Supplement: Supplementary file 4 [file Table_1.docx]

Supplementary Table I. Cytokine/Chemokine multiplex assays. *Below detectable threshold; ^---^ No detectable cytokine/chemokine; p.i.: post inoculation.

| **Cytokine/**  **chemokine** | **Time**  **p.i.** | **TgSCs**  **(pg/mL)** | **OECs**  **(pg/mL)** | **Macrophages (pg/mL)** | **McCoyB (pg/mL)** |
| --- | --- | --- | --- | --- | --- |
| IL-1α | 3 h | --- | ^*^0.43 ± 0.23 | --- | --- |
|  | 6 h | --- | --- | --- | --- |
|  | 12 h | --- | --- | --- | --- |
|  | 24 h | --- | ^*^0.55 ± 0.47 | **0.78 ± 0.40** | --- |
|  | 48 h | --- | **1.06 ± 0.49** | **2.92 ± 0.62** | --- |
| IL-4 | 3 h | *0.32 ± 0.12 | *0.28 ± 0.08 | **0.4 ± 0.10** | **0.39 ± 0.08** |
|  | 6 h | --- | *0.15 ± 0.07 | *0.21 ± 0.17 | *0.11 ± 0.04 |
|  | 12h | --- | *0.11 ± 0.07 | **0.31 ± 0.23** | *0.15 ± 0.01 |
|  | 24 h | --- | *0.26 ± 0.03 | **0.37± 0.29** | *0.33 ± 0.23 |
|  | 48 h | --- | *0.26 ± 0.06 | **1 ± 0.07** | *0.21 ± 0.04 |
| IL-5 | 3 h | --- | **0.84 ± 0.60** | --- | --- |
|  | 6 h | --- | *0.45 ± 0.51 | --- | --- |
|  | 12 h | *0.11 ± 0.09 | *0.56±0.10 | --- | --- |
|  | 24 h | *0.22 ± 0.22 | **0.97 ± 0.47** | *0.25 ± 0.26 | --- |
|  | 48 h | *0.39 ± 0.29 | *0.56 ± 0.32 | *1.11 ± 0.81 | --- |
| IL-9 | 3 h | *1.01 ± 0.28 | *2.13 ± 1.06 | *1.98 ± 0.25 | --- |
|  | 6 h | *0.23 ± 0.12 | *1.61 ± 0.77 | **0.69 ± 0.54** | *0.05 ± 0.83 |
|  | 12 h | *0.35 ± 0.07 | *1.43 ± 0.54 | *1.08 ± 0.01 | *1.26 ± 0.26 |
|  | 24 h | *0.81 ± 0.58 | *2.38 ± 0.71 | *1.80 ± 0.50 | *1.26 ± 0.26 |
|  | 48 h | *2.23 ± 0.57 | *1.68 ± 0.45 | **5.82 ± 0.92** | --- |
| IL-10 | 3 h | *2.32 ± 2.01 | **3.76 ±0.64** | **4.84 ± 2.06** | *3.01 ± 1.58 |
|  | 6 h | *0.49 ± 0.58 | *3.01±1.35 | *1.33 ± 1.87 | *0.54 ± 0.28 |
|  | 12 h | *1.06 ± 0.12 | *2.39±1.20 | *2.63 ± 2.12 | *3.76 ± 0.52 |
|  | 24 h | *3.03 ± 2.74 | **4.72 ± 2.62** | *3.95 ± 0.26 | *4.10 ± 3.11 |
|  | 48 h | **4.84 ± 2.27** | *3.75 ± 1.34 | **13.89 ± 0.74** | *3.02 ± 0.80 |
| IL-13 | 3 h | --- | --- | --- | --- |
|  | 6 h | --- | --- | --- | --- |
|  | 12 h | --- | --- | --- | --- |
|  | 24 h | --- | --- | --- | --- |
|  | 48 h | --- | --- | **11.85 ± 2.23** | --- |
| Eotaxin | 3 h | --- | **1.45 ± 0.58** | --- | --- |
|  | 6 h | --- | **1.13 ± 0.53** | --- | --- |
|  | 12 h | --- | --- | --- | --- |
|  | 24 h | **2.13 ± 0.63** | --- | --- | --- |
|  | 48 h | **1.47 ± 2.79** | --- | **6.36 ± 1.29** | --- |
| G-CSF | 3 h | *1.70 ± 1.13 | *3.35 ± 1.28 | *1.61 ± 1.14 | --- |
|  | 6 h | *0.63 ± 0.29 | *2.92 ± 0.56 | *0.67 ± 0.18 | --- |
|  | 12 h | *1.42 ± 0.56 | *3.15 ± 0.32 | *0.42 ± 0.18 | --- |
|  | 24 h | *2.24 ± 0.64 | *4.10 ± 0.98 | *1.60 ± 0.38 | --- |
|  | 48 h | *3.76 ± 3.33 | *5.37 ± 3.77 | **10.53 ± 4.21** | --- |
| RANTES | 3 h | --- | --- | --- | --- |
|  | 6 h | --- | --- | --- | --- |
|  | 12 h | --- | --- | --- | --- |
|  | 24 h | --- | **19.56 ± 6.84** | **23.02 ± 0.98** | **68.57 ± 12.54** |
|  | 48 h | **47.39 ± 21.38** | **47.49 ± 23.99** | **164.50 ± 58.87** | **102.24 ± 30.08** |
